# Bivalirudin *vs* heparin in cardiac-cerebral ischemic and bleeding events among Chinese STEMI patients during percutaneous coronary intervention: a retrospective cohort study

**DOI:** 10.1590/1414-431X2023e13013

**Published:** 2023-11-13

**Authors:** Zhichao Bai, Zhenzhen Wang, Qiang Feng, Yapei Zhang, Mengying Zhang, Aijun Hou, Yiping Wu, Zhenpeng Qin, Lina Chai

**Affiliations:** 1 Department of Cardiology, HanDan Central Hospital, Handan, China; 2 The Fourth Department of Oncology, HanDan Central Hospital, Handan, China; 3 Department of Neurology, HanDan Central Hospital, Handan, China; 4 Information Section, HanDan Central Hospital, Handan, China

**Keywords:** Bivalirudin, Heparin, Percutaneous coronary intervention, ST-segment elevation myocardial infarction, Safety profile

## Abstract

Although bivalirudin has been recently made available for purchase in China, large-scale analyses on the safety profile of bivalirudin among Chinese patients is lacking. Thus, this study aimed to compare the safety profile of bivalirudin and heparin as anticoagulants in Chinese ST-segment elevation myocardial infarction (STEMI) patients undergoing percutaneous coronary intervention (PCI). A total of 1063 STEMI patients undergoing PCI and receiving bivalirudin (n=424, bivalirudin group) or heparin (n=639, heparin group) as anticoagulants were retrospectively enrolled. The net adverse clinical events (NACEs) within 30 days after PCI were recorded, including major adverse cardiac and cerebral events (MACCEs) and bleeding events (bleeding academic research consortium (BARC) grades 2-5 (BARC 2-5)). The incidences of NACEs (10.1 *vs* 15.6%) (P=0.010), BARC 2-5 bleeding events (5.2 *vs* 10.3%) (P=0.003), and BARC grades 3-5 (BARC 3-5) bleeding events (2.1 *vs* 5.5%) (P=0.007) were lower in the bivalirudin group compared to the heparin group, whereas general MACCEs incidence (8.9 *vs* 6.4%) (P=0.131) and each category of MACCEs (all P>0.05) did not differ between two groups. Furthermore, the multivariate logistic analyses showed that bivalirudin (*vs* heparin) was independently correlated with lower risk of NACEs (OR=0.508, P=0.002), BARC 2-5 bleeding events (OR=0.403, P=0.001), and BARC 3-5 bleeding events (OR=0.452, P=0.042); other independent risk factors for NACEs, MACCEs, or BARC bleeding events included history of diabetes mellitus, emergency operation, multiple lesional vessels, stent length >33.0 mm, and higher CRUSADE score (all P<0.05). Thus, bivalirudin presented a better safety profile than heparin among Chinese STEMI patients undergoing PCI.

## Introduction

Cardiovascular diseases are the leading cause of death globally, taking the lives of an estimated 17.9 million people (1). ST-segment elevation myocardial infarction (STEMI) is viewed as the most severe manifestation of coronary artery disease and causes a great number of cardiac deaths globally (2,3). So far, the prognosis of STEMI patients is greatly improved by percutaneous coronary intervention (PCI), which is a minimally invasive treatment strategy (4,5). The PCI-related adverse events include myocardial infarction (ranging from 3.0 to 6.3%) and bleeding events (approximately 7.0%) (6,7). During PCI, anticoagulants including heparin with or without glycoprotein IIb/IIIa inhibitors (GPIs) are commonly applied (8,9). However, current anticoagulants can also cause several adverse events such as thrombocytopenia and bleeding, sometimes leading to death (10,11). Thus, research into a safe anticoagulant is imperative to improve the management of STEMI patients undergoing PCI.

Bivalirudin is an oligopeptide anticoagulant with several advantages, including direct inhibition of thrombin, rapid onset, short half-life, good safety profile, etc. (12,13). Several studies have shown that bivalirudin presents a favorable safety profile as an anticoagulant in PCI (14-17). For instance, the incidence of thrombocytopenia and bleeding is only 1.9 and 1.7%, respectively, among STEMI patients undergoing PCI and using bivalirudin as an anticoagulant (14). Moreover, the incidences of net adverse clinical events (NACEs) and cardiac death are lower among elder coronary artery disease patients receiving bivalirudin compared to those receiving heparin as an anticoagulant in PCI (15). Nevertheless, considering that bivalirudin is new in China, more large-scale analyses focused on the safety profile of bivalirudin among Chinese STEMI patients undergoing PCI are necessary to promote its clinical application.

The current study aimed to explore the incidence of and risk factors for total NACEs, major adverse cardiac and cerebral events (MACCEs), and bleeding events of bivalirudin and heparin as anticoagulants in 1063 Chinese STEMI patients receiving PCI.

## Material and Methods

### Patients

This retrospective cohort study included 1063 STEMI patients who were treated with PCI and received bivalirudin or heparin as anticoagulants in HanDan Central Hospital (China) between December 2017 and February 2022. The screening criteria were: a) diagnosed with STEMI according to the European Society of Cardiology Guidelines (18); b) over 18 years old; c) patients underwent their initial episode of PCI; d) received bivalirudin or heparin as anticoagulants. The exclusion criteria were: a) had incomplete clinical data for analysis; b) had cancer or severe hematological disease; c) were known pregnant or nursing mothers. The study was approved by the Ethics Committee of the Hospital. Written informed consent was obtained from each patient or family member.

### Treatment

Patients received bivalirudin or heparin (unfractionated heparin or low molecular weight heparin) as anticoagulants based on the current disease status, physician advice, and patient willingness. GPIs were administrated if needed. Patients who received bivalirudin were considered as the bivalirudin group (n=424) and patients who received heparin were considered as the heparin group (n=639). The regimens of bivalirudin and heparin were in accordance with a previous study (19). In the bivalirudin group, bivalirudin was administered intravenously with a loading dose of 0.75 mg/kg, then pumped continuously at a rate of 1.75 mg/kg per hour until the end of PCI and maintained for at least 30 min after the procedure. The activated clotting time (ACT) was monitored 5 min after the first dose, and if ACT was less than 225 s, additional bivalirudin was administered intravenously at 0.30 mg/kg. In the heparin group, heparin was administered intravenously with a loading dose of 80∼100 U/kg before PCI. The ACT was monitored 5 min after the first dose, and if the ACT was less than 200 s, additional heparin was administered intravenously at 20 U/kg. PCI was performed by experienced interventional cardiologists using the same equipment and standard techniques.

### Data collection

Data of STEMI patients were obtained, which included demographic characteristics, medical history, disease characteristics, and treatment information. In addition, NACEs within 30 days after PCI were recorded, which included MACCEs and bleeding events (bleeding academic research consortium (BARC) grades 2-5 (BARC 2-5)) (20,21). MACCEs contained all-cause death, recurrent myocardial infarction, ischemia-driven target vessel revascularization, and stroke (20).

### Statistical analysis

Statistics were performed using SPSS v 22.0 (IBM Corp., USA), and figures were designed by GraphPad Prism v 6.1 (GraphPad Software Inc., USA). Differences between the bivalirudin group and the heparin group were analyzed by Student's *t*-test, chi-squared test, or Wilcoxon rank sum test. Independent factors for NACEs, MACCEs, BARC 2-5 bleeding events, or BARC 3-5 bleeding events were assessed by forward stepwise multivariate logistic regression analysis with all parameters included. P<0.05 was considered significant.

## Results

### Clinical characteristics of STEMI patients

In the heparin group, the mean age of patients was 64.4±12.0 years, and there were 179 (28%) females and 460 (72%) males. In the bivalirudin group, the mean age of patients was 63.3±11.3 years, and there were 108 (25.5%) and 316 (74.5%) females and males, respectively. Moreover, no differences were found between groups in demographic characteristics, medical history, disease characteristics, and treatment information (except GPIs); the number of patients in the heparin group receiving GPIs (431 (67.4%)) was higher than those in the bivalirudin group (261 (61.6%; P=0.048) (Table 1).

**Table 1:** Clinical characteristics of STEMI patients.

Items	Heparin group(n=639)	Bivalirudin group(n=424)	P value
Demographic characteristics			
Age (years), mean±SD	64.4±12.0	63.3 ±11.3	0.155
Gender, n (%)			0.361
Female	179 (28.0)	108 (25.5)	
Male	460 (72.0)	316 (74.5)	
BMI (kg/m^2^), mean±SD	23.6±3.0	23.8±3.1	0.308
Medical history, n (%)			
Hypertension	425 (66.5)	268 (63.2)	0.268
Diabetes mellitus	133 (20.8)	92 (21.7)	0.730
Cardiac surgery	33 (5.2)	24 (5.7)	0.725
Disease characteristics			
CRUSADE score, median (IQR)	29.0 (21.0-40.0)	27.0 (19.0-38.0)	0.111
Operative timing, n (%)			0.067
Elective operation	167 (26.1)	90 (21.2)	
Emergency operation	472 (73.9)	334 (78.8)	
Infarction-related artery, n (%)			0.995
LAD	271 (42.4)	181 (42.7)	
LCX	131 (20.5)	86 (20.3)	
RCA	237 (37.1)	157 (37.0)	
Lesional vessel, n (%)			0.863
Single	545 (85.3)	360 (84.9)	
Multiple	94 (14.7)	64 (15.1)	
Treatment information			
PCI type, n (%)			0.677
Balloon dilatation	31 (4.9)	23 (5.4)	
Stent implantation	608 (95.1)	401 (94.6)	
Stent diameter (mm), median (IQR)	3.0 (3.0-3.5)	3.0 (2.8-3.5)	0.321
Stent length (mm), median (IQR)	33.0 (23.0-38.0)	33.0 (23.0-38.0)	0.401
GPIs, n (%)	431 (67.4)	261 (61.6)	0.048

STEMI: ST-segment elevation myocardial infarction; BMI: body mass index; CRUSADE: Can Rapid Risk Stratification of Unstable Angina Patients Suppress Adverse Outcomes with Early Implementation of the ACC/AHA guidelines; IQR: interquartile range; LAD: left anterior descending artery; LCX: left circumflex artery; RCA: right coronary artery; PCI: percutaneous coronary intervention; GPIs: glycoprotein IIb/IIIa inhibitors. Student's *t*-test, chi-squared test, or Wilcoxon rank sum test.

### Comparison of the safety profile of heparin and bivalirudin

**Figure 1:**
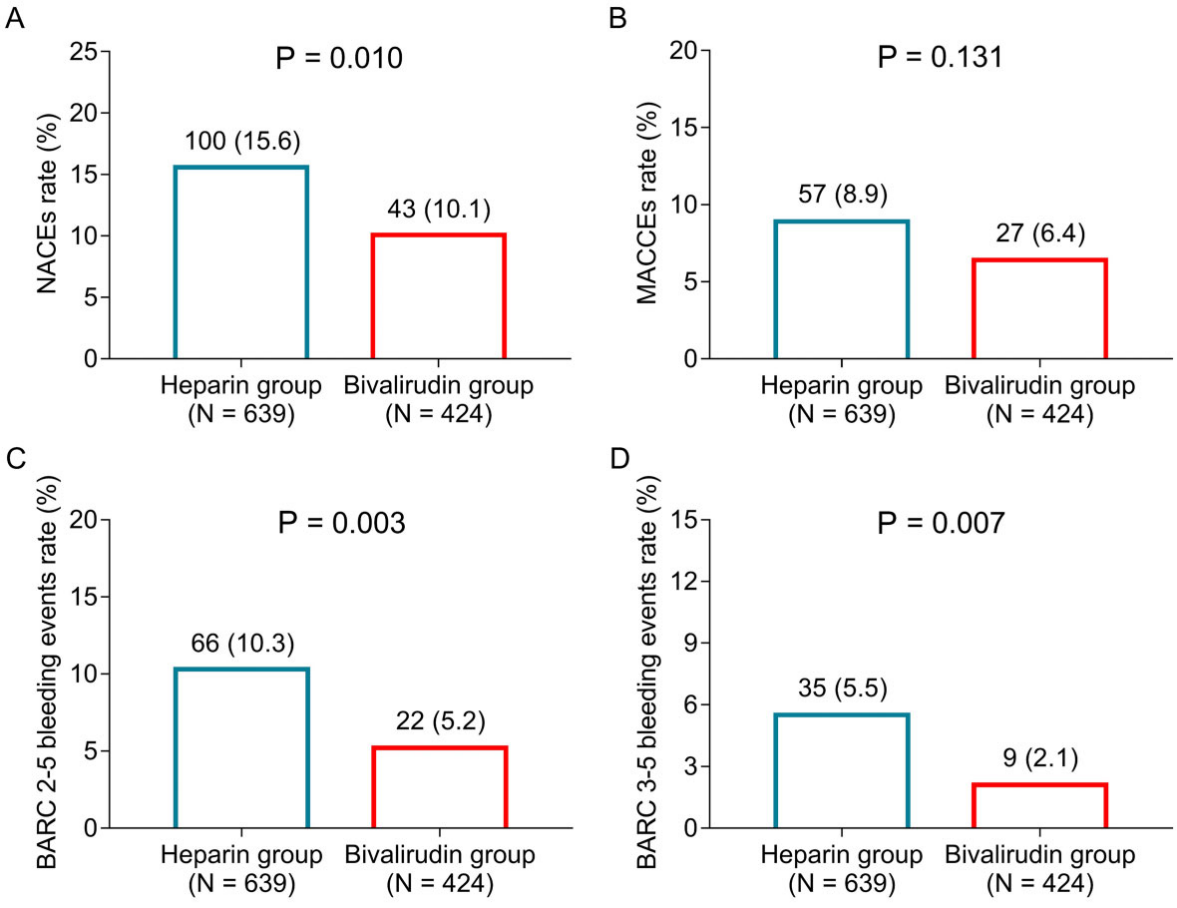
Safety profile in the heparin and bivalirudin groups among ST-segment elevation myocardial infarction (STEMI) patients undergoing percutaneous coronary intervention. Comparison of the incidence of net adverse clinical events (NACEs) (**A**), major adverse cardiac and cerebral events (MACCEs) (**B**), bleeding academic research consortium (BARC) 2-5 scores (**C**), and BARC 3-5 scores (**D**). Numbers on the top of the columns are number and percentage (Student’s *t*-test).

The NACEs rate was lower in the bivalirudin group compared to the heparin group (10.1 *vs* 15.6%) (P=0.010) (Figure 1A). However, no difference in the MACCEs rate was found between the two groups (8.9 *vs* 6.4%) (P=0.131) (Figure 1B). Furthermore, the rate of BARC 2-5 bleeding events was lower in the bivalirudin group compared to the heparin group (5.2 *vs* 10.3%) (P=0.003) (Figure 1C). A lower rate of BARC 3-5 bleeding events was also found in the bivalirudin group (2.1 *vs* 5.5%) (P=0.007) (Figure 1D). To further explore the differences in MACCEs between the heparin group and the bivalirudin group, the incidence rate of each event of MACCEs was recorded, and the analysis showed that all-cause mortality, cardiac mortality, recurrent myocardial infarction, ischemia-driven revascularization, and stroke did not differ between groups (all P>0.05) (Table 2).

**Table 2:** Detailed MACCEs of STEMI patients.

Items	Heparin group(n=639)	Bivalirudin group(n=424)	P value
All-cause mortality, n (%)	32 (5.0)	20 (4.7)	0.830
Cardiac mortality, n (%)	23 (3.6)	17 (4.0)	0.731
Recurrent myocardial infarction, n (%)	15 (2.3)	5 (1.2)	0.170
Ischemia-driven revascularization, n (%)	13 (2.0)	8 (1.9)	0.866
Stroke, n (%)	10 (1.6)	6 (1.4)	0.844

MACCEs: major adverse cardiac and cerebral events; STEMI: ST-segment elevation myocardial infarction. Student’s *t*-test.

### Factors related to NACEs

Patients who received bivalirudin (*vs* heparin) as treatment had a lower risk of NACEs (odds ratio (OR)=0.508, P=0.002), while history of diabetes mellitus (*vs* no) (OR=1.818, P=0.007), emergency operation (*vs* elective operation) (OR=2.700, P<0.001), multiple (*vs* single) lesional vessels (OR=2.030, P=0.006), and stent length >33.0 mm (*vs* ≤33.0 mm) (OR=1.550, P=0.026) were associated with a higher risk of NACEs (Table 3).

**Table 3:** Factors associated with NACEs.

Items	P value	OR	95%CI	
			Lower	Upper
Treatment (bivalirudin *vs* heparin)	0.002	0.508	0.334	0.774
History of diabetes mellitus (yes *vs* no)	0.007	1.818	1.180	2.799
Operative timing (emergency operation *vs* elective operation)	<0.001	2.700	1.579	4.617
Lesional vessel (multiple *vs* single)	0.006	2.030	1.224	3.368
Stent length (>33.0 *vs* ≤33.0 mm)	0.026	1.550	1.054	2.278

NACEs: net adverse clinical events; OR: odds ratio; CI, confidence interval. Chi-squared test.

### Independent factors related to MACCE

A history of diabetes mellitus (*vs* no) was the only factor independently correlated with a higher number of MACCEs (OR=1.995, P=0.007).

### Factors related to BARC 2-5 bleeding events

Bivalirudin (*vs* heparin) as treatment (OR=0.403, P=0.001) was related to a lower risk of BARC 2-5 bleeding events, while history of diabetes mellitus (*vs* no) (OR=1.854, P=0.021), emergency operation (*vs* elective operation) (OR=2.180, P=0.017), multiple (*vs* single) lesional vessels (OR=1.958, P=0.032), and stent length >33.0 mm (*vs* ≤33.0 mm) (OR=1.840, P=0.014) were associated with an increased risk of BARC 2-5 bleeding events (Table 4).

**Table 4:** Factors associated with BARC 2-5 bleeding events.

Items	P value	OR	95%CI	
			Lower	Upper
Treatment (bivalirudin *vs* heparin)	0.001	0.403	0.231	0.700
History of diabetes mellitus (yes *vs* no)	0.021	1.854	1.096	3.136
Operative timing (emergency operation *vs* elective operation)	0.017	2.180	1.152	4.124
Lesional vessel (multiple *vs* single)	0.032	1.958	1.061	3.613
Stent length (>33.0 *vs* ≤33.0 mm)	0.014	1.840	1.133	2.989

BARC 2-5 bleeding events: Bleeding Academic Research Consortium grades 2 to 5; OR: odds ratio; CI: confidence interval. Chi-squared test.

### Factors related to BARC 3-5 bleeding events

Bivalirudin (*vs* heparin) as treatment (OR=0.452, P=0.042) was associated with a lower risk of BARC 3-5 bleeding events, while a higher CRUSADE score (OR=3.799, P<0.001) and stent length >33.0 mm (*vs* ≤33.0 mm) (OR=2.361, P=0.014) were associated with a higher risk of BARC 3-5 bleeding events (Table 5).

**Table 5:** Factors associated with BARC 3-5 bleeding events.

Items	P value	OR	95% CI	
			Lower	Upper
Treatment (bivalirudin *vs* heparin)	0.042	0.452	0.211	0.970
CRUSADE score (high *vs* low)	<0.001	3.799	1.989	7.259
Stent length (>33.0 *vs* ≤33.0 mm)	0.014	2.361	1.191	4.677

BARC 3-5 bleeding events: Bleeding Academic Research Consortium grades 3 to 5; OR: odds ratio; CI: confidence interval; CRUSADE: Can Rapid Risk Stratification of Unstable Angina Patients Suppress Adverse Outcomes with Early Implementation of the ACC/AHA guidelines. Chi-squared test.

## Discussion

The current study found that: 1) the incidences of NACE, BARC 2-5 bleeding events, and BARC 3-5 bleeding events were lower in the bivalirudin group compared to the heparin group; 2) bivalirudin (*vs* heparin) was independently associated with lower risk of NACE, BARC 2-5 bleeding events, and BARC 3-5 bleeding events. This study indicated that bivalirudin presented a better safety profile than heparin among Chinese STEMI patients undergoing PCI.

Our findings are in agreement with several previous studies. For example, in the EUROMAX trial, the rate of major bleeding was minimal with bivalirudin (5.1%), followed by heparin (7.6%) and heparin plus GPI (9.8%) among STEMI patients undergoing PCI (22). Moreover, stent thrombosis was lower with bivalirudin (0.4%) compared to heparin (0.7%) among acute myocardial infarction (AMI) patients undergoing PCI in Sweden (23). Importantly, one large-scale prospective study from China explored the 30-day adverse events and adverse drug reactions of bivalirudin as an anticoagulant among Chinese AMI patients undergoing PCI and showed that bivalirudin had a good safety profile (14).

The findings of the current study might be due to the fact that heparin indirectly suppresses the activity of thrombin while bivalirudin is a direct thrombin inhibitor. In addition, bivalirudin reduced platelet activity compared to heparin (12,13,24), showing a better capability of inhibiting major bleeding events, which account for a large part of NACEs. In addition, the current study found no difference in overall MACCEs incidence and single MACCEs between STEMI patients receiving bivalirudin and heparin in the current study, which was consistent with previous studies (25,26). Bivalirudin might not be able to directly affect cardiac and cerebral function while directly inhibiting bleeding events.

To further help clinicians improve the management of STEMI patients undergoing PCI, the risk factors of NACEs, MACCEs, BARC 2-5 bleeding events, and BARC 3-5 bleeding events were also explored in the current study. Bivalirudin (*vs* heparin) was associated with a lower risk of NACEs, BARC 2-5 bleeding events, and BARC 3-5 bleeding events; other factors associated with NACEs, MACCEs, or BRAC bleeding events included history of diabetes mellitus, emergency operation, multiple lesional vessels, stent length >33.0 mm, and higher CRUSADE score, which was partly in line with the previous studies (14,26,27). Clinicians should be aware of STEMI patients receiving PCI with the above risk factors.

The clinical implications of the current study are: 1) bivalirudin as an anticoagulant during PCI might reduce the risk of severe bleeding complications in STEMI patients, which is especially crucial during high-risk procedures like PCI; 2) bivalirudin was associated with fewer NACEs compared to heparin. The lower rate of NACEs indicated that using bivalirudin might result in better overall patient outcomes; 3) for STEMI patients undergoing PCI who were at a higher risk of complications, such as those with a history of bleeding or other medical conditions that increase the risk of adverse events, bivalirudin might be a suitable anticoagulant option. Evaluating clinical events within a specific time frame such as 30 days ensured safety, compliance with regulations, proper maintenance, cost-effectiveness, environmental care, quality control, and adherence to insurance requirements. BARC 2-5 bleeding events included any overt sign of hemorrhage, while BARC 3-5 bleeding events included overt bleeding plus a hemoglobin drop of 3 to 5 g/dL or intracranial hemorrhage (severe bleeding) (28).

Several limitations in the current study should not be ignored: 1) although this was a large-scale research, the low incidence of NACEs, MACCEs, and bleeding events led to low statistical power; 2) the retrospective and single-center nature of the study; 3) the short follow-up; 4) the different number of patients in the bivalirudin and heparin groups.

In conclusion, bivalirudin exhibited a better tolerance compared to heparin among Chinese STEMI patients undergoing PCI.

## References

[1] Lavie CJ (2022). Progress in cardiovascular diseases statistics 2022. Prog Cardiovasc Dis.

[2] Vogel B, Claessen BE, Arnold SV, Chan D, Cohen DJ, Giannitsis E (2019). ST-segment elevation myocardial infarction. Nat Rev Dis Primers.

[3] Frampton J, Devries JT, Welch TD, Gersh BJ (2020). Modern management of ST-segment elevation myocardial infarction. Curr Probl Cardiol.

[4] de Boer MJ, Ottervanger JP, Van't Hof AWJ, Hoorntje JCA, Suryapranata H, Zijlstra F (2022). Final benefit of primary percutaneous coronary intervention for ST-elevation myocardial infarction in older patients: long-term results of a randomised trial. Neth Heart J.

[5] Saada M, Kobo O, Polad J, Halabi M, AJJ IJ, Puentes A (2022). Prognosis of PCI in AMI setting in the elderly population: outcomes from the multicenter prospective e-ULTIMASTER registry. Clin Cardiol.

[6] Wang HY, Wang Y, Yin D, Gao RL, Yang YJ, Xu B (2020). Percutaneous coronary intervention complexity and risk of adverse events in relation to high bleeding risk among patients receiving drug-eluting stents: insights from a large single-center cohort study. J Interv Cardiol.

[7] Leonardi S, Lopes RD, Steg PG, Abnousi F, Menozzi A, Prats J (2018). Implications of different criteria for percutaneous coronary intervention-related myocardial infarction on study results of three large phase III clinical trials: The CHAMPION experience. Eur Heart J Acute Cardiovasc Care.

[8] Giustino G, Colombo A, Camaj A, Yasumura K, Mehran R, Stone GW (2022). Coronary in-stent restenosis: JACC state-of-the-art review. J Am Coll Cardiol.

[9] Bainey KR, Marquis-Gravel G, Mehta SR, Tanguay JF (2022). The evolution of anticoagulation for percutaneous coronary intervention: a 40-year journey. Can J Cardiol.

[10] Piran S, Schulman S (2019). Treatment of bleeding complications in patients on anticoagulant therapy. Blood.

[11] Patriarcheas V, Pikoulas A, Kostis M, Charpidou A, Dimakakos E (2020). Heparin-induced thrombocytopenia: pathophysiology, diagnosis and management. Cureus.

[12] Taylor T, Campbell CT, Kelly B (2021). A review of bivalirudin for pediatric and adult mechanical circulatory support. Am J Cardiovasc Drugs.

[13] Erdoes G, Ortmann E, De Arroyabe BML, Reid C, Koster A (2020). Role of bivalirudin for anticoagulation in adult perioperative cardiothoracic practice. J Cardiothorac Vasc Anesth.

[14] Zheng H, Wang Z, Li Q, Zhao Y, Liu Y, Chen A (2022). Comprehensive safety profile evaluation of bivalirudin in Chinese ST-segment elevation myocardial infarction patients receiving percutaneous coronary intervention: a prospective, multicenter, intensive monitoring study. BMC Cardiovasc Disord.

[B15] Li J, Liu X, Ma S, Na K, Qi Z, Xu Y (2022). Effectiveness and safety of bivalirudin in elderly patients with coronary artery disease undergoing percutaneous coronary intervention: a real-world study. Catheter Cardiovasc Interv.

[B16] Qaderdan K, Vos GJA, McAndrew T, Steg PG, Hamm CW, Van't Hof A (2017). Outcomes in elderly and young patients with ST-segment elevation myocardial infarction undergoing primary percutaneous coronary intervention with bivalirudin versus heparin: pooled analysis from the EUROMAX and HORIZONS-AMI trials. Am Heart J.

[17] Fabris E, Kilic S, Van't Hof AWJ, Ten Berg J, Ayesta A, Zeymer U (2017). One-Year mortality for bivalirudin vs heparins plus optional glycoprotein iib/iiia inhibitor treatment started in the ambulance for ST-segment elevation myocardial infarction: a secondary analysis of the EUROMAX randomized clinical trial. JAMA Cardiol.

[18] Ibanez B, James S, Agewall S, Antunes MJ, Bucciarelli-Ducci C, Bueno H (2018). 2017 ESC Guidelines for the management of acute myocardial infarction in patients presenting with ST-segment elevation: The Task Force for the management of acute myocardial infarction in patients presenting with ST-segment elevation of the European Society of Cardiology (ESC). Eur Heart J.

[19] Chen S, Li Y, Qiu M, Jiang Z, Han Y, Li J (2021). Comparison of the effects of heparin and bivalirudin on percutaneous coronary intervention in female patients with coronary heart disease [in Chinese]. Clin J Med Offic.

[20] Ki YJ, Lee BK, Park KW, Bae JW, Hwang D, Kang J (2022). Prasugrel-based De-escalation of dual antiplatelet therapy after percutaneous coronary intervention in patients with STEMI. Korean Circ J.

[21] Chen H, Yu X, Kong X, Li L, Wu J, Ma L (2020). Efficacy and safety of bivalirudin application during primary percutaneous coronary intervention in older patients with acute ST-segment elevation myocardial infarction. J Int Med Res.

[22] Zeymer U, Van't Hof A, Adgey J, Nibbe L, Clemmensen P, Cavallini C (2014). Bivalirudin is superior to heparins alone with bailout GP IIb/IIIa inhibitors in patients with ST-segment elevation myocardial infarction transported emergently for primary percutaneous coronary intervention: a pre-specified analysis from the EUROMAX trial. Eur Heart J.

[23] Erlinge D, Omerovic E, Frobert O, Linder R, Danielewicz M, Hamid M (2017). Bivalirudin *versus* heparin monotherapy in myocardial infarction. N Engl J Med.

[24] Fu D, Liu M, Gao T, Li C, Liao J, Shao M (2021). Evaluation of bivalirudin-associated major adverse cardiac and hemorrhagic events in acute coronary syndrome patients on chronic dialysis following percutaneous coronary intervention. J Invasive Cardiol.

[25] Lopes RD, Alexander KP, Manoukian SV, Bertrand ME, Feit F, White HD (2009). Advanced age, antithrombotic strategy, and bleeding in non-ST-segment elevation acute coronary syndromes: results from the ACUITY (Acute Catheterization and Urgent Intervention Triage Strategy) trial. J Am Coll Cardiol.

[26] Venetsanos D, Lawesson SS, James S, Koul S, Erlinge D, Swahn E (2018). Bivalirudin *versus* heparin with primary percutaneous coronary intervention. Am Heart J.

[27] Li P, Zhang H, Luo C, Ji Z, Zheng Z, Li Z (2022). Occurrence and risk factors of adverse drug reactions in patients receiving bivalirudin as anticoagulant during percutaneous coronary intervention: a prospective, multi-center, intensive monitoring study. Front Cardiovasc Med.

[28] (2019). Dual Antiplatelet Therapy following percutaneous coronary intervention: clinical and economic impact of standard versus extended duration - recommendations.

